# Lack of effectiveness of Bebtelovimab monoclonal antibody among high-risk patients with SARS-Cov-2 Omicron during BA.2, BA.2.12.1 and BA.5 subvariants dominated era

**DOI:** 10.1371/journal.pone.0279326

**Published:** 2023-04-28

**Authors:** Srilekha Sridhara, Ahmet B. Gungor, Halil K. Erol, Mohanad Al-Obaidi, Tirdad T. Zangeneh, Edward J. Bedrick, Venkatesh K. Ariyamuthu, Aneesha Shetty, Abd A. Qannus, Katherine Mendoza, Sangeetha Murugapandian, Gaurav Gupta, Bekir Tanriover

**Affiliations:** 1 Division of Nephrology, College of Medicine, The University of Arizona, Tucson, Arizona, United States of America; 2 Division of Nephrology, The Banner University Medical Center, Tucson, Arizona, United States of America; 3 Division of Infectious Diseases, College of Medicine, The University of Arizona, Tucson, Arizona, United States of America; 4 Department of Epidemiology and Biostatistics, College of Public Health, The University of Arizona, Tucson, Arizona, United States of America; 5 Division of Nephrology, Virginia Commonwealth University, Richmond, Virginia, United States of America; SUNY Upstate Medical University: State University of New York Upstate Medical University, UNITED STATES

## Abstract

Severe acute respiratory syndrome coronavirus 2 (SARS-CoV-2) Omicron subvariants are expected to be resistant to Bebtelovimab (BEB) monoclonal antibody (MAb) and the real-world experience regarding its effectiveness is scarce. This retrospective cohort study reports a data analysis in Banner Healthcare System (a large not-for-profit organization) between 4/5/2022 and 8/1/2022 and included 19,778 Coronavirus disease-19 (COVID-19) positive (by PCR or direct antigen testing) patients who were selected from Cerner-Electronic Health Record after the exclusions criteria were met. The study index date for cohort was determined as the date of BEB MAb administration or the date of the first positive COVID-19 testing. The cohort consist of COVID-19 infected patients who received BEB MAb (N = 1,091) compared to propensity score (PS) matched control (N = 1,091). The primary composite outcome was the incidence of 30-day all-cause hospitalization and/or mortality. All statistical analyses were conducted on the paired (matched) dataset. For the primary composite outcome, the event counts and percentages were reported. Ninety-five percent Clopper-Pearson confidence intervals for percentages were computed. The study cohorts were 1:1 propensity matched without replacement across 26 covariates using an optimal matching algorithm that minimizes the sum of absolute pairwise distance across the matched sample after fitting and using logistic regression as the distance function. The pairs were matched exactly on patient vaccination status, BMI group, age group and diabetes status. Compared to the PS matched control group (2.6%; 95% confidence interval [CI]: 1.7%, 3.7%), BEB MAb use (2.2%; 95% CI: 1.4%, 3.3%) did not significantly reduce the incidence of the primary outcome (p = 0.67). In the subgroup analysis, we observed similar no-difference trends regarding the primary outcomes for the propensity rematched BEB MAb treated and untreated groups, stratified by patient vaccination status, age (<65 years or ≥65), and immunocompromised status (patients with HIV/AIDS or solid organ transplants or malignancy including lymphoproliferative disorder). The number needed to treat (1/0.026–0.022) with BEB MAb was 250 to avoid one hospitalization and/or death over 30 days. The BEB MAb use lacked efficacy in patients with SARS-CoV-2 Omicron subvariants (mainly BA.2, BA.2.12.1, and BA.5) in the Banner Healthcare System in the Southwestern United States.

## Introduction

Coronavirus disease 2019 (COVID19), caused by severe acute respiratory syndrome coronavirus 2 (SARS-CoV-2), presents with a spectrum of infection severity, ranging from asymptomatic to severe/critical [1]. Disease severity is classified as mild to moderate (viral symptoms [2] and no or mild pneumonia), severe (hypoxia requiring oxygen support and/or >50% lung involvement on imaging), and critical (respiratory failure, multiorgan failure, shock) [1, 3]. The individual risk of severity varies by age, underlying medical conditions, vaccination status, and SARS-CoV-2 variants [4, 5].

SARS-CoV-2 continues to evolve into new variants of concern (VOC) characterized by mainly spike receptor binding domain mutations, which are the target of authorized neutralizing monoclonal antibodies (MAb) to reduce hospitalization and death [6]. The spike protein mutations of SARS-CoV-2 Omicron subvariants have reduced susceptibility to earlier authorized MAbs (e.g. bamlanivimab-etesevimab, casirivimab-imdevimab, and sotrovimab) for outpatient treatment of coronavirus disease-19 (COVID-19) [6–10]. Based on invitro and limited clinical data [11], the Food and Drug Administration (FDA) granted Emergency Use Authorization (EUA) for LY-CoV1404 (Bebtelovimab [BEB]) on February 11, 2022, as an alternative therapy for high-risk patients with mild to moderate COVID-19 [9].

In our system, the Banner Healthcare System, a multidisciplinary team (Monoclonal Antibody Treatment Program) reviews patients’ eligibility for antiviral therapy (remdesivir and nirmatrelvir-ritonavir as the first line agents) and an alternative MAb treatment, guided by the FDA EUA. BEB is indicated for high-risk patients at risk for severe disease and/or death who are unable to receive remdesivir 3-days IV treatment due to logistic challenges or have contraindications for the use of nirmaltrevir/ritonavir due to severe drug-drug interactions. The high-risk patients with underlying medical conditions are defined by the Center for Disease Control and Prevention (CDC), such as age>50 years old, obesity (body mass index [BMI] >30, hematological malignancies, solid organ recipients, human immunodeficiency virus (HIV) infection, chronic lung/ kidney/ liver diseases, etc. [12]. BEB was recommended based on laboratory results indicating potent activity against the Omicron VOC and other VOCs based on data from the Phase 2 BLAZE-4 study [9, 11]. However, there is still no phase 3 clinical trial data to support BEB’s use, and real-world experience is limited in the Omicron subvariants dominated era [13, 14].

In this study, we assessed the composite outcome (all-cause hospitalization and/or death over 30-day) in high-risk patients in the outpatient setting, who received BEB MAb compared to the propensity score (PS) matched untreated control group for COVID-19 in the Banner Healthcare System (a large not-for-profit organization) in the Southwestern United States, during a period (4/5/2022-8/1/2022) dominated by SARS-CoV-2 Omicron BA.2, BA.2.12.1, and BA.5 subvariants [15]. We primarily used publicly available CDC’s National SARS-CoV-2 Genomic Surveillance System database (https://covid.cdc.gov/covid-data-tracker/#variant-proportions) and CoVariants database (https://covariants.org/per-country?region=United+States) to decide on prevalent Omicron subvariants in the areas of U.S. where Banner Healthcare System operates during the study period, not based on the viral genotype sampling from the study cohort.

## Methods

### Patient consent statement

This study was approved by the Institutional Review Board of the University of Arizona with a waiver of patient consent given the retrospective nature of the study. The study adhered to the Strengthening the Reporting of Observational Studies in Epidemiology (STROBE) statement (See S1 File).

### Overview

This observational retrospective cohort study of positive COVID-19 patients was conducted between April 5, 2022, and August 1, 2022. Patients’ follow-up date was censored on August 31, 2022. All data pertaining to BEB MAb treated patients and untreated patients were captured from electronic health records (Cerner EHR) in the Banner Health Care System, which houses thirty hospitals and several clinics across the Southwestern United States, mainly in Arizona. A multidisciplinary team formed under the Banner Health Care System Monoclonal Antibody Treatment program reviews patients’ eligibility for antiviral therapy (remdesivir and nirmatrelvir-ritonavir as the first line agents) and an alternative MAb treatment, guided by the FDA EUA. The alternative BEB MAb therapy (175 mg administered as a single intravenous injection over 30 seconds) is indicated for mild-to-moderate SARS-CoV-2 infection (within 7 days of symptom onset) in adults who are at high-risk for progression to severe disease and in children older than 12 years-old and weighing 40 kg or above.

**Fig 1:**
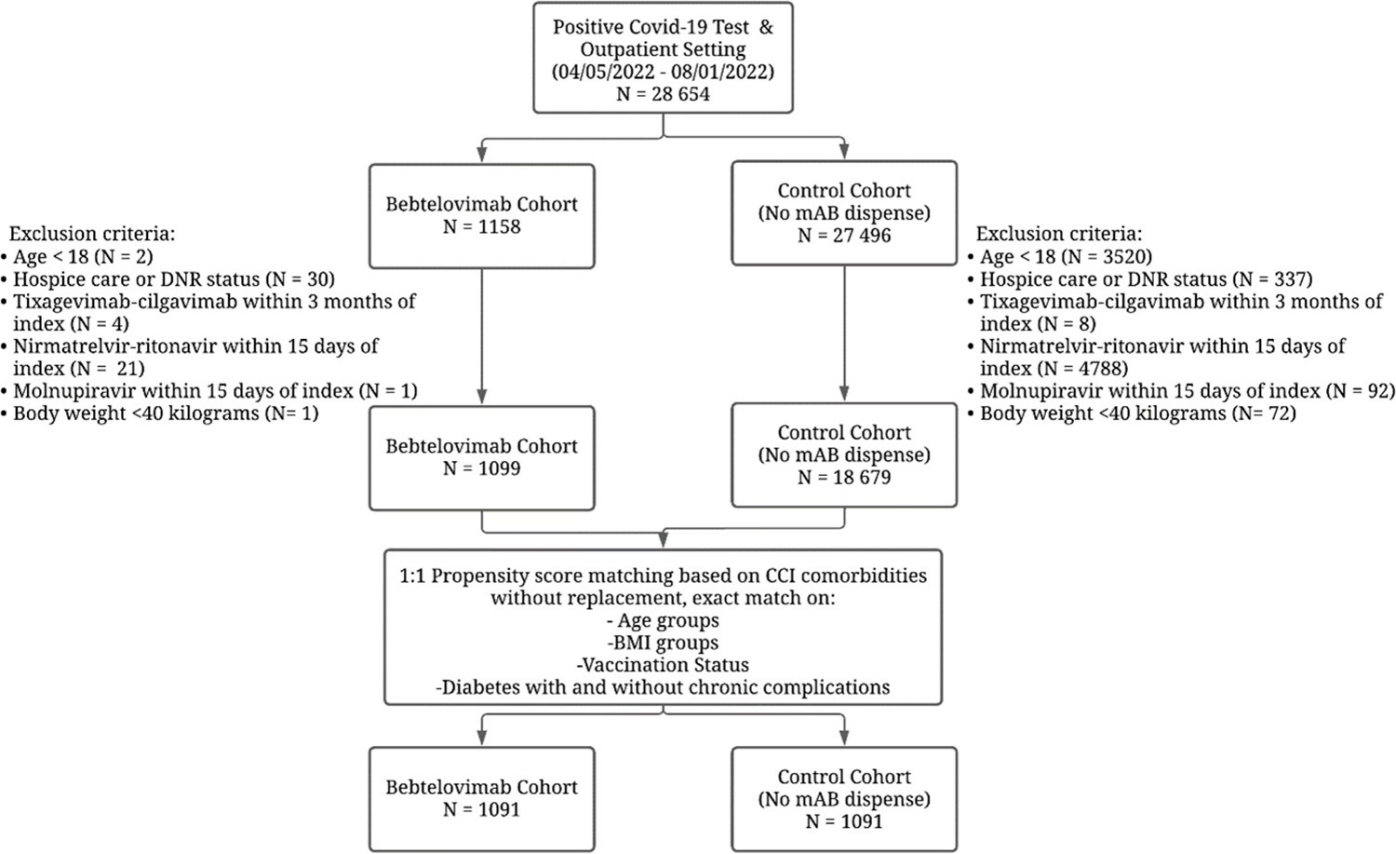
Study cohort selection.

During the study period, 19,778 COVID-19 positive (by PCR or direct antigen testing) patients were retrieved from Cerner-EHR after exclusions were met (Fig 1) and were split into BEB Mab treated and untreated control cohort. Patients were excluded if they were younger than 18 years of age, in hospice care, received BEB MAb in the inpatient setting, received tixagevimab-cilgavimab prophylactic MAb (Evusheld) within last 3 months/ nirmatrelvir-ritonavir (Paxlovid) within 15 days/ molnupiravir (Lagevrio) within 15 days of index date, or weighted less than 40 kilograms. During the study period, there were 12 MAB infusion sites (for the treatment cohort) and 128 testing sites in the Banner Health Care System. The study index date for cohorts was determined as the date of BEB MAb administration or the date of the first positive COVID-19 testing. Index dates were used as an enrollment date for the study. Demographic and clinical covariates of both cohorts were extracted from the EHR. Clinical covariates were derived from the Charlson Comorbidity Index codes (based on International Classification of Diseases, Tenth Revision [ICD-10] codes documented in the EHR within five years preceding the patient index date). The resulting pre-propensity matched study cohort comprised 1,099 BEB MAb treated patients and 18,679 untreated patients with 26 covariates to be included in the propensity model. One-to-one propensity score matching with no replacement was performed to match both cohorts. The matched cohort consisted of 1,091 pairs (N = 2,182 patients).

### Outcomes

The primary composite outcome was the incidence of all-cause hospitalization and/or mortality and the within 30-days of the index date among the post-propensity matched cohort.

### Propensity score matching

Propensity score methods (matching, stratification, covariate adjustment, and inverse probability of treatment weighting) are commonly used in non-randomized observational data analysis to balance covariate differences between treatment groups and reduce bias in estimating treatment effects (pseudo-randomization) [16]. The goal is to enable investigators to generate similar study groups to measure the relationship more accurately between treatment and outcome of interest. Propensity score is the probability of receiving a treatment of interest based on participants’ comorbidities, treating provider’s preference, clinic setting/cluster. These probabilities are then applied to adjust for differences between groups. To assess balance post-propensity matched groups, standardized mean differences (SMD-difference in mean outcome between groups divided by standard deviation of outcome among participants) are commonly used and SMD <0.1 generally indicates negligible difference (in other words optimal matching).

The cohorts in this study were 1:1 propensity matched without replacement across 26 covariates using an optimal matching algorithm (a logistic regression model) that minimizes the sum of absolute pairwise distance across the matched sample. Optimal matching is a generalized linear model with a logit link function to calculate propensity scores. MatchIt package from the statistical computing software R was used for optimal pair matching [17]. This algorithm was chosen over the nearest neighbors and complete matching algorithms due to its better performance in balancing the covariates in between the treated and untreated cohort and for the lesser number of unmatched patients. The pairs were matched exactly on patient vaccination status, BMI group, age group and diabetes status (with and without complications). This meant that, for example, a fully vaccinated BEB MAb treated patient could only be matched with a fully vaccinated untreated patient. Patients were classified as fully vaccinated if they had at least two or three (depending on immunocompromised status) COVID-19 mRNA technology vaccine (Pfizer or Moderna) reported in the EHR. The vaccination status of Arizona residents is available through a web-portal (the Arizona State Immunization Information System) [18]. Alongside the exact matched variables, all the demographic and clinical covariates outlined in Table 1 as well as a calculated date variable (composed of one-month periods between the study start and end date; accounts for possible monthly difference in prevalent Omicron subvariants) were included as predictors in a logistic regression model to estimate the propensity score under the optimal matching algorithm. Distance function was determined based on its ability to keep covariate standardized mean differences (SMDs) to a minimum while keeping the matched patients at the maximum level. The covariate balance was assessed by comparing these pre- and post-matches SMDs with a covariate balance plot and by looking at empirical CDF statistics for each covariate.

**Table 1:** Patient characteristics and covariate balance before and after propensity matching.

	After Propensity Matching			Before Propensity Matching		
	BEB Treatment Cohort	Untreated Control Cohort	SMD	BEB Treatment Cohort	Untreated Control Cohort	SMD
	N = 1,091	N = 1,091		N = 1,099	N = 18,679	
Age	64.0 [50.0,74.0]	64.0 [50.0,74.0]		64.0 [50.0,74.0]	46.0 [32.0,63.0]	
Age Groups						
18–35	82 (7.5)	82 (7.5)	0.00	83 (7.6)	5,969 (32.0)	-0.92
35–50	200 (18.3)	200 (18.3)	0.00	202 (18.4)	4,568 (24.5)	0.16
50–60	168 (15.4)	168 (15.4)	0.00	169 (15.4)	2,849 (15.3)	0.00
60–70	275 (25.2)	275 (25.2)	0.00	275 (25.0)	2,539 (13.6)	0.26
>70	366 (33.5)	366 (33.5)	0.00	370 (33.7)	2,754 (14.7)	0.40
Sex						
Male	469 (43.0)	474 (43.4)	-0.01	474 (43.1)	7,366 (39.4)	0.07
Fully Vaccinated						
Yes	748 (68.6)	748 (68.6)	0.00	752 (68.4)	7,430 (39.8)	0.62
No	298 (27.3)	298 (27.3)	0.00	301 (27.4)	5,538 (29.6)	-0.05
Unknown	45 (4.1)	45 (4.1)	0.00	46 (4.2)	5,711 (30.6)	-1.32
Race/Ethnicity						
White	859 (78.7)	866 (79.4)	-0.02	867 (78.9)	11,801 (63.2)	0.39
Black	48 (4.4)	44 (4.0)	0.02	48 (4.4)	1,058 (5.7)	-0.06
Hispanic	120 (11.0)	109 (10.0)	0.03	120 (10.9)	3,542 (19.0)	-0.26
Asian/Pacific Islander	13 (1.2)	16 (1.5)	-0.03	13 (1.2)	360 (1.9)	-0.07
Native American/Alaskan	8 (0.7)	9 (0.8)	-0.01	8 (0.7)	228 (1.2)	-0.06
Unknown	43 (3.9)	47 (4.3)	-0.02	43 (3.9)	1,690 (9.0)	-0.26
BMI Group						
≤20	24 (2.2)	24 (2.2)	0.00	26 (2.4)	811 (4.3)	-0.13
20–25	164 (15.0)	164 (15.0)	0.00	166 (15.1)	4,005 (21.4)	-0.18
25–30	305 (28.0)	305 (28.0)	0.00	307 (27.9)	4,968 (26.6)	0.03
30–35	236 (21.6)	236 (21.6)	0.00	236 (21.5)	3,477 (18.6)	0.07
35–40	160 (14.7)	160 (14.7)	0.00	161 (14.6)	1,844 (9.9)	0.14
>40	137 (12.6)	137 (12.6)	0.00	137 (12.5)	1,545 (8.3)	0.13
Unknown	165 (6.0)	65 (6.0)	0.00	66 (6.0)	2,029 (10.9)	-0.20
Time period						
4/05-30/2022	103 (9.4)	131 (12.0)	-0.09	103 (9.4)	1,213 (6.5)	0.10
5/01-31/2022	249 (22.8)	268 (24.6)	0.00	252 (22.9)	3,652 (19.6)	0.08
6/01-30/2022	372 (34.1)	358 (32.8)	0.04	375 (34.1)	6,746 (36.1)	-0.04
7/01-31/2022	367 (33.6)	334 (30.6)	0.03	369 (33.6)	7,040 (37.7)	-0.09
8/01/2022	0 (0.0)	0 (0.0)	0.00	0 (0.0)	28 (0.1)	-0.04
Myocardial Infarction	64 (5.9)	47 (4.3)	0.06	65 (5.9)	526 (2.8)	0.13
Heart Failure	91 (8.3)	73 (6.7)	0.06	93 (8.5)	797 (4.3)	0.15
Cerebrovascular Disease	93 (8.5)	66 (6.0)	0.09	94 (8.6)	674 (3.6)	0.18
Hemiplegia or Paraplegia	12 (1.1)	11 (1.0)	0.01	12 (1.1)	161 (0.9)	0.02
Peripheral Vascular Disease	100 (9.2)	84 (7.7)	0.05	101 (9.2)	725 (3.9)	0.18
Chronic Pulmonary Disease	343 (31.4)	311 (28.5)	0.06	346 (31.5)	3,907 (20.9)	0.23
Dementia	21 (1.9)	19 (1.7)	0.01	21 (1.9)	237 (1.3)	0.05
Hypertension	573 (52.5)	534 (48.9)	0.07	580 (52.8)	5,199 (27.8)	0.50
Diabetes without Chronic Complications	242 (22.2)	242 (22.2)	0.00	246 (22.4)	2,236 (12.0)	0.25
Diabetes with Chronic Complications	104 (9.5)	104 (9.5)	0.00	110 (10.0)	838 (4.5)	0.18
Renal Mild-Moderate-Advanced Disease (CKD stage 1–4)	147 (13.5)	118 (10.8)	0.08	153 (13.9)	780 (4.2)	0.28
Renal Severe Disease (CKD stage 5 and ESRD)	37 (3.4)	21 (1.9)	0.08	39 (3.5)	201 (1.1)	0.13
Mild Liver Disease	117 (10.7)	95 (8.7)	0.07	117 (10.6)	1,034 (5.5)	0.17
Moderate to Severe Liver Disease	34 (3.1)	18 (1.6)	0.08	35 (3.2)	185 (1.0)	0.13
Peptic Ulcer Disease	26 (2.4)	18 (1.6)	0.05	26 (2.4)	224 (1.2)	0.08
Rheumatic Disease	60 (5.5)	57 (5.2)	0.01	60 (5.5)	466 (2.5)	0.13
Malignancy, skin cancers and lymphoproliferative disorders	84 (7.7)	72 (6.6)	0.04	84 (7.6)	645 (3.5)	0.16
Metastatic Solid Tumor	25 (2.3)	25 (2.3)	0.00	25 (2.3)	148 (0.8)	0.10
HIV/AIDS/Opportunistic Infections	120 (11.0)	94 (8.6)	0.08	123 (11.2)	1,425 (7.6)	0.11
Solid Organ Transplant	53 (4.9)	31 (2.8)	0.09	56 (5.1)	150 (0.8)	0.20

Data are presented as mean [SD] for continuous measures, and n (%) for categorical measures.

Abbreviations: MAb = monoclonal antibody; SMD = standardized mean difference; IQR = interquartile range; BMI = body mass index; CKD = chronic kidney disease; ESRD = end-stage renal disease; HIV = Human Immunodeficiency Virus; AIDS = acquired immunodeficiency syndrome.

### Statistical analysis

**Fig 2:**
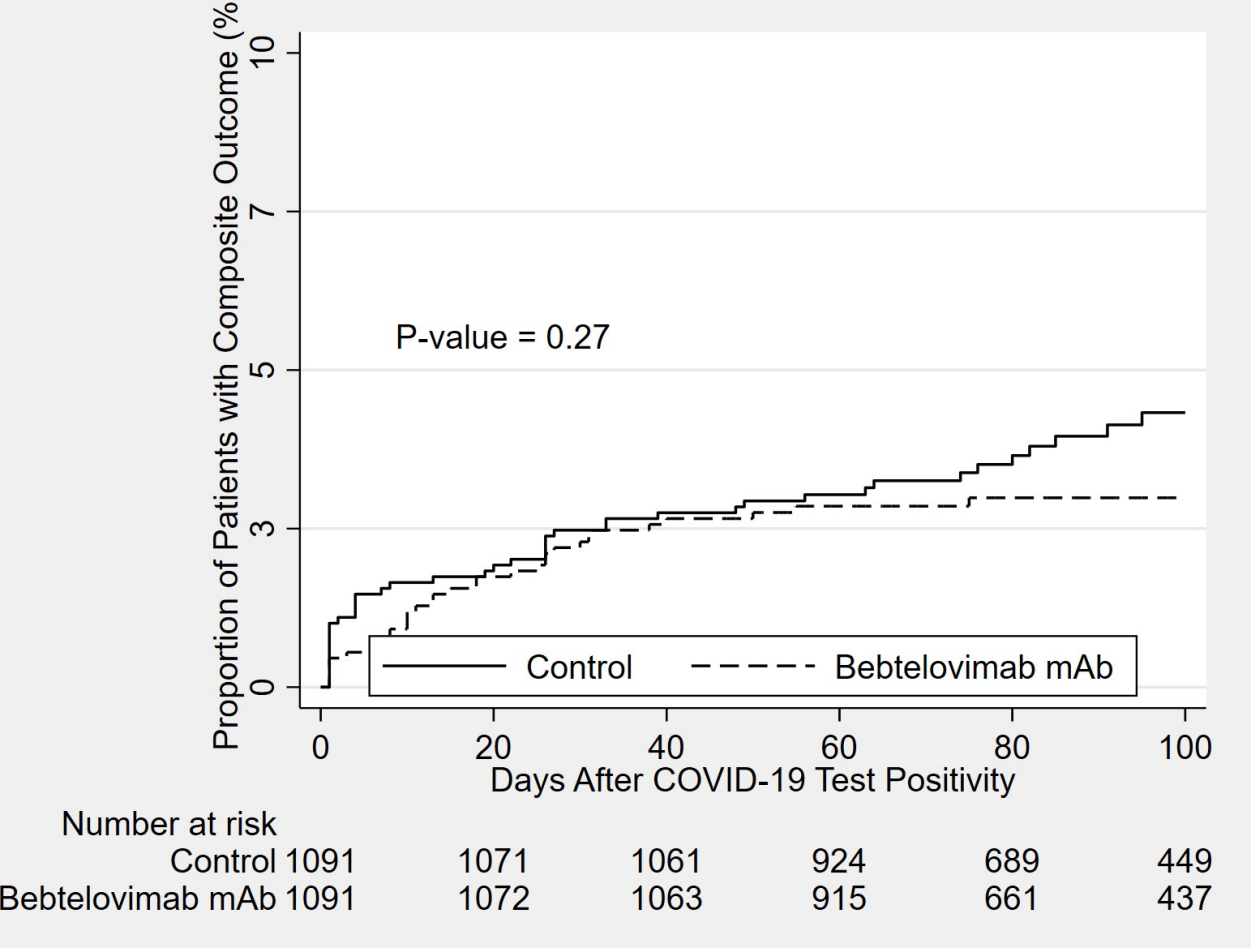
Kaplan Meier curves for the composite outcome in patients who received Bebtelovimab monoclonal antibody treated group vs. not-treated control group between April 5, 2022, and August 1, 2022.

All statistical analyses were conducted on the paired (matched) dataset. For each outcome, event count, percentage with the event and ninety-five percent Clopper-Pearson confidence intervals have been reported. These confidence intervals for percentages were computed using the R package Exactci. Exact McNemar’s test was used to compare the proportions in the paired dataset and the 95% confidence intervals for proportions were calculated. P-values <0.05 was considered statistically significant (Table 2). Additionally, the Kaplan-Meier estimator was used to plot curves for the primary composite outcome between the post-PS matched groups during the study period (Fig 2).

**Table 2:** The primary composite outcome between the propensity matched Bebtelovimab (BEB) monoclonal antibody (MAb) and untreated control groups.

Primary outcomes in post-propensity score-matched cohorts						
	BEB MAb Treatment Group		Untreated Control Group			
	N (%)	95% CI*	N (%)	95% Cl*	Difference in % with 95% CI**	*P-value*
Composite outcome within 30 days						
Whole cohort	24 (2.2)	1.4, 3.3	28 (2.6)	1.7, 3.7	-0.4 (-1.7, 1.0)	0.67
All-cause hospitalization within 30 days						
Whole cohort	24 (2.2)	1.4, 3.3	27 (2.5)	1.6, 3.6	-0.3 (-1.6, 1.1)	0.77
Mortality within 30 days						
Whole cohort	0 (0.0)	0.0, 0.3	3 (0.3)	0.1, 0.8	-0.3 (-0.8, 0.1)	0.25

Abbreviations: BEB = Bebtelovimab; MAb = monoclonal antibody; CI = Confidence Interval.

* The Clopper-Pearson method was used to calculate 95% confidence intervals for the outcome percentages using the R package (Exactci).

** CI for difference in paired proportions between the treatment and control cohorts.

### Subgroup analysis

Additional analyses were conducted on subgroups under the propensity matched cohorts to assess the differences on BEB MAb effectiveness on these subgroups. For this purpose, the post-match BEB treated and untreated cohorts were combined and then stratified by vaccination status (fully vaccinated and not fully vaccinated), age groups (age <65 and age ≥65), and immunocompromised status (patients with comorbidities including HIV/AIDS, malignancy, solid organ transplantation, and patients without these comorbidities). The same algorithm and model that were used for the initial propensity matching were employed to rematch based on subgroups and the incidence of the composite outcome was reported alongside the percent difference in between the treated and untreated cohorts and its significance level (Table 3). Finally, we constructed a Cox proportional hazard model to predict the hazards for the primary composite outcome in the PS matched subgroups (BEB MAb use yes/no, fully vaccinated yes/no, immunocompromised yes/no, and age groups age <65 or ≥65) (Table 4).

**Table 3:** Subgroup analysis for the primary composite outcome stratified by patient vaccination status (fully vaccinated vs. not fully vaccinated), age category (age <65 vs. age ≥65), and immunocompromised status (comorbidities including HIV/AIDS or solid organ transplants or malignancy) between the propensity matched Bebtelovimab (BEB) and untreated control groups.

Primary outcome in the post-propensity-matched study cohort with subgroups						
	BEB MAb Treatment Group		Untreated Control Group			
	N (%)	95% CI*	N (%)	95% Cl**	Difference in % with 95% CI***	*P-value*
Fully vaccinated* N = 1,496	7 (0.9)	0.4, 1.9	11 (1.5)	0.7, 2.6	-0.5 (-1.8, 0.7)	0.48
Not fully vaccinated N = 596	17 (5.7)	3.4, 9.0	17 (5.7)	3.4, 9.0	0.0 (-4.0, 4.0)	1.00
Immunocompromised N = 250	9 (7.2)	3.3, 13.2	11 (8.8)	4.5, 15.2	-1.6 (-9.2, 6.0)	0.81
Not- Immunocompromised N = 1,636	10 (1.2)	0.6, 2.2	7 (0.9)	0.3, 1.8	0.4 (-0.8, 1.5)	0.63
Age ≥65 N = 1,014	13 (2.6)	1.4, 4.3	17 (3.4)	2.0, 5.3	-0.8 (-3.1, 1.5)	0.57
Age <65 N = 1,068	8 (1.5)	0.6, 2.9	9 (1.7)	0.8, 3.2	-0.2 (-2.0, 1.6)	1.00

Abbreviations: HIV = human immunodeficiency virus; AIDS = acquired immunodeficiency syndrome; Bebtelovimab = BEB; MAb = monoclonal antibody; CI = Confidence Interval.

*This analysis included the patients with known vaccination status only.

**The Clopper-Pearson method was used to calculate 95% confidence intervals (CI) for the outcome percentages using the R package (Exactci).

*** CI for difference in paired proportions between the BEB MAb treatment and control cohorts.

**Table 4:** Multivariable Cox proportional hazard model* for the composite outcome among the post-propensity matched COVID-19 infected patients in the Banner Healthcare System between April 5, 2022, and August 1, 2022.

	Hazard Ratio (95% Confidence Interval)	Standard Error	P-value
Bebtelovimab monoclonal antibody use (yes vs. no)	0.75 (0.43–1.31)	0.21	0.31
Fully vaccinated status (Yes vs. no)	0.23 (0.12-.42)	0.07	<0.001
Age (≥65 vs. <65)	2.07 (1.15–3.74)	2.41	0.02
Immunocompromised** (Yes vs. no)	4.60 (2.58–8.19)	5.19	<0.001

*Accounted for the paired data.

**Immunocompromised status (the patients with HIV/AIDS or solid organ transplants or malignancy including lymphoproliferative disorder).

### Missing data

Data on missing vaccination status of patients was labeled as unknown and was included in the initial propensity matching (Table 1).

## Results

### Patient characteristics

Table 1 shows the characteristics of BEB MAb and untreated control cohorts before and after propensity matching. All post-propensity matching covariate SMDs were < 0.1 threshold, indicating an optimal matching. In the post propensity matched cohort, the median age of patients in the BEB MAb treatment group was 64 (interquartile range [IQR], 50–74) years; 43% were male, and 78.7% were White race and 68.6% patients were fully vaccinated. Some of the high-risk characteristics included age ≥60 years (58.7%), hypertension [52.5%], diabetes mellitus (31.7%), chronic pulmonary disease (31.4%), BMI ≥35 kg/m2 (27.3%), chronic kidney disease–any stage (16.9%), chronic liver disease (13.8%), human immunodeficiency virus infection (HIV/AIDS) and/or opportunistic infections (11%), heart failure (8.3%), malignancy including lymphoproliferative disease (7.7%), and solid organ transplant and hematopoietic stem cell transplants (4.9%).

### Outcomes

Table 2 shows the result of the primary composite outcome within 30 days in the post propensity matched cohorts. Compared to the untreated control group, the incidence of patients with the composite outcome in the BEB MAb treated group within 30 days is 2.2% (95%: CI 1.4% to 3.3%) vs. 2.6% (95% CI: 1.7% to 3.7%) (P-value = 0.67). The all-cause hospitalizations within 30 days in the BEB MAb cohort was 2.2% (95% CI: 1.4% to 3.3%) vs 2.5% (95% CI, 1.6% to 3.6%) (P value = 0.77); the proportion of patients with all-cause mortality within 30 days was 0% (95% CI, 0% to 0%) vs 0.3% (95% CI, 0.1% to 0.8%; P-value = 0.25). Fig 2 showed no difference between the Kaplan Meier curves for the primary composite outcome stratified by BEB MAb treatment status at last follow-up (P-value = 0.27). The number needed to treat (1/0.026–0.022) with BEB MAb was 250 to avoid one hospitalization and/or death over 30 days.

In the subgroup analysis, we observed similar no-difference trends regarding the primary composite outcomes for the propensity rematched BEB MAb treated and untreated groups, stratified by patient vaccination status, age (<65 years or ≥65), and immunocompromised status (patients with HIV/AIDS or solid organ transplants or malignancy including lymphoproliferative disorder), see Table 3 below.

## Hazard model for primary composite outcome among the propensity matched cohorts

Table 4 shows the multivariable Cox proportional hazards model, (accounted for the paired data) predicting hazards for the composite outcome among the post-PS patients. The BEB MAb use was not associated with statistically significant lower hazards of composite outcome (hazard ratio [HR] 0.75; 95% CI: 0.43 to 1.31, P-value = 0.31). However, fully vaccinated status continued to be protective while age >65 and immunosuppressed status increased the hazards for primary outcome two to four folds, respectively.

## Discussion

In this retrospective propensity matched analysis, the incidence of the primary composite outcome was low (2.2%-2.6%) and treatment with the BEB MAb lacked efficacy against SARS-CoV-2 Omicron during an era dominated by BA.2, BA.2.12.1, and BA.5 subvariants to reduce the all-cause hospitalization and mortality over 30 days in Banner Health Care System in the Southwestern United States. Moreover, in the subgroup analysis for the primary composite outcome stratified by patient vaccination status, age category, and immunocompromised status between the PS matched groups, BEB MAb use failed to show significant efficacy. The hazards for the composite outcome were lower in the BEB MAb group but not statistically significant. However, fully vaccinated status continued to be protective while age >65 and immunosuppressed status increased the hazards for primary outcome two to four folds, respectively. Similar finding from epidemiological study showing possible protective immunity from previous infections and vaccinations, and that older age can result in worse outcomes during the Omicron wave [19]. Such findings can help stratifying risk groups when administering COVID-19 therapeutics.

The only published (non-peer-reviewed data) on the efficacy of BEB MAb comes from the Phase II Blaze 4 clinical trial during the period of alpha and delta waves, which showed that the incidence of the primary outcome (hospitalization or death over 29 days) in the BEB MAb arm compared with the control arm was similar, around 3% [11]. While in vitro studies showing preserved neutralization of SARS-COV2 variants (BA.2 and BA. 5) [20, 21], it was not demonstrated in clinical trials. Hence, the real-world experience with BEB Mab use, especially during the periods of new variants emergence, is limited to a couple of recently published studies in the general population [13, 14] and solid organ transplant cohorts [22, 23]. A Mayo clinic study (N = 2,833) reported that the BEB MAb use was associated with very low incidence of the primary outcome (1.4%, 95% CI: 1.2% to 1.7%) between 3/20/2022 and 6/14/2022, dominated by Omicron BA.2 subvariant [14]. However, the study was limited by a population of predominantly white and fully vaccinated (>90%) patients, and moreover, the study lacked a matched control group and did not clearly define exclusion criteria (e.g., tixagevimab-cilgavimab prophylactic MAb (Evusheld) use etc.). Therefore, in the absence of control group comparison, it is difficult to ascertain the author’s conclusion of primary outcome of 1.4% because of the fully vaccinated status of patients or the effect of the BEB MAb use. In contrast, our data suggest that the fully vaccinated patients had similar primary outcome of 0.9% in BEB MAb group vs. 1.5% in non-treated PS matched group, which signifies the importance of population immunity. In another study (N = 930 patients in each arm), the University of Pittsburgh researchers showed that BEB MAb use, between 3/30/2022 and 5/30/2022, significantly reduced 30-day hospitalization and/or death compared to the PS matched cohort, 3.1% vs. 5.5%, respectively [13]. But the protective effect was the most prominent among older, immunosuppressed and fully vaccinated patients. The authors did not exclude the patients who received tixagevimab-cilgavimab prophylactic MAb (Evusheld) and the cohort included small proportions of racial minorities (Blacks/Hispanics/Asians) comprised approximately 4% of the final study cohort. The SARS-Cov-2 Omicron BA.2 subvariant dominated the COVID-19 infections during that study period. In terms of SOT recipients who received the BEB MAb, another study from the Mayo Clinic [23] reported 3.3% incidence of the primary outcome in their cohort (N = 92) and a smaller number of SOT recipients received BEB MAb compared to Sotrovimab MAb group. In contrast, the SOT recipients in our cohort (n = 53) had much higher incidence of hospitalization and death (9.4% in BEB MAb group vs. 6.5% in control arm, P-value = 0.64). The differences in vaccination rates and the different effects of Omicron subvariants may account for this variation.

On November 10, 2022, the NIH COVID-19 Treatment Guidelines Panel [24] reported that certain rapidly increasing Omicron subvariants (e.g., BQ.1 and BQ.1.1) are likely to be resistant to BEB MAb [25] based on in vitro neutralization studies [26]. The panel recommend BEB MAb only as an alternative treatment for when preferred ritonavir-boosted nirmatrelvir or remdesivir are not available or contraindicated, and when most of the spreading (>50%) Omicron subvariants in a given region are susceptible, the rating of evidence rated as level III (expert opinion). Later, on November 30, 2022, the U.S. Food & Drug Administration removed the EUA of BEB because a non-susceptible SARS-CoV-2 subvariants account for majority of COVID 19 cases (Omicron BQ.1 and BQ.1.1 subvariants infections to be above 57% nationally) [27].

Human genetic predisposition (host high-risk Human Leukocyte Antigen haplotypes, higher expression of angiotensin converting enzyme polymorphisms and cellular proteases [e.g. Furin and TMPRSS2 involved in viral entry and infectivity]) can explain why some individuals are severely affected upon infection by SARV-CoV-2 resulting in severe inflammatory lung injury [28–30]. The GenOMICC (Genetics of Mortality in Critical Care) study utilizing whole-genome sequencing compared the genomes of 7,491 patients with critical COVID19 with 48,400 controls and discovered 16 new high-risk variants within genes involved in interferon signaling, leucocyte differentiation and blood-type antigen secretion which are critically important in uncontrolled viral replication, enhanced pulmonary inflammation, and intravascular thrombosis [31]. These findings are very critical to identify disease mechanism, risk stratification and new targets for drug development. Unfortunately, we do not have human genome analysis data in our cohort, nor it is a routine practice to obtain whole genome sequencing for risk stratification. It is difficult for us to predict its effect on cohort selection and the result of this study since we do not know the distribution of high-risk genetic variants in the Southwestern U.S.

Observational data analysis in the post-propensity matched cohort cannot be used to prove causality nor propensity score methods can eliminate unmeasured confounding as randomize controlled trials do. Quality of propensity score adjustment depends on propensity model, selection of variables, and sample size. If the propensity model is appropriately constructed and sample size large enough, propensity score methods can approximate treatment effects accurately. By using optimal matching algorithm, a large sample size. very small SMDs (<0.1) across 26 covariates, we feel confident that our results reflect a reasonable approximation of BEB treatment effect.

Large observational (real-life experiences) studies are necessary to show the efficacy assessment of MAbs in the setting of continuously mutating SARS-Cov-2 when conducting conventional randomized controlled clinical trials may not be practical. However, our study has several limitations: 1) retrospective study design not allowing to rule residual confounding; 2) lack of symptom severity assessment among patients (possibility of more symptomatic patients on the BEB MAb group vs. asymptomatic patients on the control arm); 3) not measuring the impact of immunity through prior COVID-19 infection(s); 4) not knowing patients’ vaccination booster status (3^rd^ or 4^th^ booster); 5) lack of specific SARS-Cov-2 Omicron subvariant genotype sampling; 6) not capturing patients may have received Ritonavir-boosted nirmatrelvir (Paxlovid) and other approved therapies by healthcare providers outside our healthcare system.

In conclusion, the BEB MAb use lacked efficacy in patients with SARS-CoV-2 Omicron subvariants in the Banner Healthcare System (a large not-for-profit organization) in the Southwestern United States. Under the light of the current study findings and an expectation of the majority of Omicron subvariants becoming resistant, the continuing use of BEB MAb may no longer be justified. Continuing real-world research from other large healthcare organizations in the different regions of the United States would be needed to assess generalizability.

## Supporting information

S1 File STROBE PLOS one. (PDF)
