# Supplementary material for: Lack of effectiveness of Bebtelovimab monoclonal antibody among high-risk patients with SARS-Cov-2 Omicron during BA.2, BA.2.12.1 and BA.5 subvariants dominated era
Source: PLoS One. 2023 Apr 28;18(4):e0279326. doi: 10.1371/journal.pone.0279326 (PMC10146441; doi:10.1371/journal.pone.0279326)
Supplement: S1 File — (PDF) [file pone.0279326.s001.pdf]

Modified STROBE Statement—checklist of items that should be included in reports of observational studies (Cohort Sstudies)

	Item No	Recommendation	Authors' Statement
**Title and abstract**	1	(*a*) Indicate the study's design with a commonly used term in the title or the abstract	(Abstract) "a retrospective cohort study regarding comparative effectiveness of Bebtelovimab (BEB) monoclonal antibody (MAb) use"
		(*b*) Provide in the abstract an informative and balanced summary of what was done and what was found	(Abstract) "counts, percentages, and confidence intervals are provided; the BEB MAb use lacked efficacy in patients with SARS-CoV-2 Omicron subvariants compared to control"
**Introduction**			
Background/rationale	2	Explain the scientific background and rationale for the investigation being reported	(Intro) describes lack of Phase III trials and real world experiences regarding effectiveness of Beblovimab (BEB) in era of SARS-CoV-2 Omicron subvariants.
Objectives	3	State specific objectives, including any prespecified hypotheses	(Intro) "Comparison of all-cause hospitalization and/or death over 30-day in high-risk outpatients, who received BEB MAb compared to the propensity score (PS) matched untreated control group for COVID-19 dominated by SARS-CoV-2 Omicron BA.2, BA.2.12.1, and BA.5 subvariants"
**Methods**			
Study design	4	Present key elements of study design early in the paper	1:1 propensity matched without replacement across 26 covariates using an optimal matching algorithm
Setting	5	Describe the setting, locations, and relevant dates, including periods of recruitment, exposure, follow-up, and data collection	Method/Overview "This observational retrospective cohort study of positive COVID-19 patients was conducted between April 5, 2022, and August 1, 2022. Patients' follow-up date was censored on August 31, 2022. All data pertaining to BEB MAb treated patients and untreated patients were captured from electronic health records (Cerner EHR) in the Banner Health Care System, which houses thirty hospitals and several clinics across the Southwestern United States, mainly in Arizona ."

Participants	6	(*a*) *Cohort study*—Give the eligibility criteria, and the sources and methods of selection of participants. Describe methods of follow-up<br><br>*Case-control study*—Give the eligibility criteria, and the sources and methods of case ascertainment and control selection. Give the rationale for the choice of cases and controls<br><br>*Cross-sectional study*—Give the eligibility criteria, and the sources and methods of selection of participants	Cohort study, as described in Methods, Flow Chart (Figure 1), and follow-up summary (Table 1).
Variables	7	Clearly define all outcomes, exposures, predictors, potential confounders, and effect modifiers. Give diagnostic criteria, if applicable	All-cause hospitalization and death within 30 days of index date, as detailed in methods. The exposure variable (BEB MAb use) clearly called out. The BEB MAb and control group were propensity matched based on 26 variables in the EHR, explained in Methods, the covariant balance pre and post-PS match was assessed (Table 1). The study conserved sample size/power for main effect estimation; effect modification was not performed.
Data sources/ measurement	8*	For each variable of interest, give sources of data and details of methods of assessment (measurement).	Clinical covariates were derived from the Charlson Comorbidity Index codes (based on International Classification of Diseases, Tenth Revision [ICD-10] codes) documented in the Cerner-EHR within five years preceding the index date.
Bias	9	Describe any efforts to address potential sources of bias	To reduce selection bias associated with the decision to administer BEB MAb, we utilized a 1:1 propensity matched without replacement across 26 covariates using an optimal matching algorithm that minimizes the sum of absolute pairwise distance across the matched sample after fitting and using logistic regression as the distance function.
Study size	10	Explain how the study size was arrived at (if applicable)	99.3% of patients who received BEB MAb were included in the post-PS analysis. This cohort was chosen to allow at least 30 days follow up during a period (4/5/2022-8/1/2022) dominated by SARS-CoV-2 Omicron BA.2, BA.2.12.1, and BA.5 subvariants.

Quantitative variables	11	Explain how quantitative variables were handled in the analyses. If applicable, describe which groupings were chosen and why	Both continuous variables and parameterization of categorical variables are explained Method section.
Statistical methods	12	(*a*) Describe all statistical methods, including those used to control for confounding	The Methods section articulate our optimal propensity matching method, exact McNemar's test comparing the proportions in the pair dataset and 95% confidence intervals.
		(*b*) Describe any methods used to examine subgroups and interactions	We also fitted a multivariable Cox proportional hazard regression model predicting the composite outcome in the PS matched subgroups. The Kaplan-Meier estimator was used to plot curves for the composite outcome between the post-PS matched groups.
		(*c*) Explain how missing data were addressed	The vaccination status was missing for 4.1% of the patients for both groups in the post-propensity matched cohort (Table 1). Missing vaccination status was analyzed as a separate category (fully vaccinated, not fully vaccinated, unknown (missing).
		(*d*) *Cohort study*—If applicable, explain how loss to follow-up was addressed	We assumed that all patients in the study cohort were followed in the Banner Healthcare system and follow-up was complete. We stated the possibility of primary outcome out-of-Banner system hospitalization as a limitation in the Discussion section.

		Case-control study—If applicable, explain how matching of cases and controls was addressed<br><br>*Cross-sectional study*—If applicable, describe analytical methods taking account of sampling strategy	*N/A*
		(*e*) Describe any sensitivity analyses	*N/A*
**Results**			
Participants	13*	(a) Report numbers of individuals at each stage of study—eg numbers potentially eligible, examined for eligibility, confirmed eligible, included in the study, completing follow-up, and analyzed	See Flow Chart (Figure 1).
		(c) **Use of a flow diagram**	See Flow Chart (Figure 1)
Descriptive data	14*	(a) Give characteristics of study participants (eg demographic, clinical, social) and information on exposures and potential confounders	Tables 1 include summary statistics of both exposures and confounders.

		(b) Indicate number of participants with missing data for each variable of interest	Included in Table 1
		(c) *Cohort study*—Summarise follow-up time (eg, average and total amount)	Included in Table 1. Each patient had at least 30 days follow-up post-index date.
Outcome data	15*	*Cohort study*—Report numbers of outcome events or summary measures over time	Included in Table 2 and Table3.
		Case-control study—Report numbers in each exposure category, or summary measures of exposure	*N/A*
		Cross-sectional study—Report numbers of outcome events or summary measures	*N/A*
Main results	16	(*a*) Give unadjusted estimates and, if applicable, confounder-adjusted estimates and their precision (eg, 95% confidence interval). Make clear which confounders were adjusted for and why they were included	Comparative proportions along with confidence intervals in the post-propensity score matched cohort and their re-matched subgroups in Table 2-3.
Other analyses	17	Report other analyses done—eg analyses of subgroups and interactions, and sensitivity analyses	Subgroup analysis for vaccinated status, age (65 or below and >65), immunosuppressed status were described and shown in Table 3. Sensitivity analysis was not performed and interaction effects were not tested.
**Discussion**			
Key results	18	Summarise key results with reference to study objectives	Description of results aligns with stated objective of estimating independent effects of the BEB MAb use.

Limitations	19	Discuss limitations of the study, taking into account sources of potential bias or imprecision. Discuss both direction and magnitude of any potential bias	Limitations, including retrospective study design, unmeasured confounding, prior COVID -19 exposures, lack of viral genotyping, and others are discussed extensively in the Discussion section.
Interpretation	20	Give a cautious overall interpretation of results considering objectives, limitations, multiplicity of analyses, results from similar studies, and other relevant evidence	Caution is incorporated into the discussion and detailed in the limitations section.
Generalisability	21	Discuss the generalisability (external validity) of the study results	Results are clearly framed as being generalizable only to Southwestern U.S. Further real-world research from large healthcare organizations in different regions of the U.S. would be needed to assess generalizability.

*Give information separately for cases and controls in case-control studies and, if applicable, for exposed and unexposed groups in cohort and cross-sectional studies.

Note: An Explanation and Elaboration article discusses each checklist item and gives methodological background and published examples of transparent reporting. The STROBE checklist is best used in conjunction with this article (freely available on the Web sites of PLoS Medicine at http://www.plosmedicine.org/, Annals of Internal Medicine at http://www.annals.org/, and Epidemiology at http://www.epidem.com/). Information on the STROBE Initiative is available at www.strobe-statement.org.
